# Incidence of Atrial High-Rate Episodes in Chagas Disease Patients

**DOI:** 10.5935/abc.20180060

**Published:** 2018-04

**Authors:** Emanoela Lima Freitas, Elieusa e Silva Sampaio, Roque Aras

**Affiliations:** Universidade Federal da Bahia, Salvador, BA - Brazil

**Keywords:** Atrial Fibrillation/complications, Cardiac Pacing, Artificial/methods, Defibrillators, Implantable, Chagas Disease/complications, Risk, Stroke/etiology

Atrial high-rate episodes (AHRE) have been described as subclinical atrial fibrillation (AF), characterized by the occurrence of atrial arrhythmias (including AF and atrial flutter) with atrial rate greater than 190 bpm^1^ or 250 bpm,^2^ duration of 5-6 minutes or longer, asymptomatic, and detected by continuous monitoring, particularly with the use of implantable cardiac electronic devices (ICEDs). There is evidence that AHRE are associated with 2-2.5 times increased risk for stroke.^3^ Although the incidence of AHRE may reach 70%,^4^ this number decreases to 30%^1^ when AF patients using oral anticoagulants are excluded. However, there are no data in the literature on AHRE incidence in some specific populations, vulnerable to thromboembolic complications, such as patients with Chagas Disease (CD). The incidence of AHRE was investigated in a cohort study with 67 Chagasic patients with ICEDs, conducted in the arrhythmia outpatient service of the University Hospital in Salvador, Brazil, between May 2016 and June 2017. Patients with AF or atrial flutter, and patients using anticoagulants were excluded. The ICEDs were set to detect AHRE episodes of atrial rate ≥ 190 bpm and duration ≥ 6 minutes, and patients were monitored for a mean period of 98 days (± 28.8 days). Mean age was 63.6 years (± 9.2 years), 67.2% were women and 50.7% were black. Cardiac pacemaker was the most common ICED (92.5%); 89.4% had the cardiac form of CD and mean ejection fraction was 58.5% (± 14.1%). The incidence of AHRE was 11.9% (8 patients) in this population. Duration of the episodes were variable among patients - 6-29min (1 patient), 30min-5h59min (5 patients), 6h-23h59min (1 patient). In one patient, the longest episode was greater than 24 hours. The mean time period for the first AHRE was 27.6 days (± 26.9). Evidence of the incidence of AHRE in Chagasic population aligns with the evidence of AHRE in other populations and gives support to the development of specific approaches involving antithrombotic therapy, until results of studies with anticoagulants become available.
